# The Causality between Human Immunoglobulin G (IgG) N-Glycosylation and Aging: A Mendelian Randomization Study

**DOI:** 10.3390/molecules29061281

**Published:** 2024-03-14

**Authors:** Wenxin Sun, Xuening Jian, Jie Zhang, Xiaoni Meng, Haotian Wang, Deqiang Zheng, Lijuan Wu, Youxin Wang

**Affiliations:** 1 School of Public Health, Capital Medical University, Beijing 100069, China; sunwx_ada@163.com (W.S.); jian_0628@126.com (X.J.); zhangjie@ccmu.edu.cn (J.Z.); mengxiaoni385@163.com (X.M.); sdwanghaotian@126.com (H.W.); deqiangzheng@163.com (D.Z.); wujuan811017@163.com (L.W.); 2 Beijing Key Laboratory of Clinical Epidemiology, Capital Medical University, Beijing 100069, China; 3 School of Public Health, North China University of Science and Technology, Tangshan 063210, China; 4 Centre for Precision Medicine, Edith Cowan University, Perth 6027, Australia

**Keywords:** IgG N-glycosylation, immune aging, Mendelian randomization, frailty index, leukocyte telomere length

## Abstract

Background: Immunoglobulin G (IgG) N-glycosylation is considered a potential biomarker for aging and various pathological conditions. However, whether these changes in IgG N-glycosylation are a consequence or a contributor to the aging process remains unclear. This study aims to investigate the causality between IgG N-glycosylation and aging using Mendelian randomization (MR) analysis. Methods: We utilized genetic variants associated with IgG N-glycosylation traits, the frailty index (FI), and leukocyte telomere length (LTL) from a previous genome-wide association study (GWAS) on individuals of European ancestry. Two-sample and multivariable MR analyses were conducted, employing the inverse-variance weighted (IVW) method. Sensitivity analyses were performed to assess potential confounding factors. Results: Using the IVW method, we found suggestive evidence of a causal association between GP14 and FI (β 0.026, 95% CI 0.003 to 0.050, *p* = 0.027) and LTL (β −0.020, 95% CI −0.037 to −0.002, *p* = 0.029) in the two-sample MR analysis. In the multivariable MR analysis, suggestive evidence was found for GP23 and FI (β −0.119, 95% CI −0.219 to −0.019, *p* = 0.019) and GP2 and LTL (β 0.140, 95% CI 0.020 to 0.260, *p* = 0.023). Conclusions: In conclusion, our results supported a potentially causal effect of lower GP23 levels on an advanced aging state. Additional verification is required to further substantiate the causal relationship between glycosylation and aging.

## 1. Introduction

N-glycosylation is an important and common posttranslational modification in which oligosaccharides are attached to specific asparagine residues of the protein backbone [1]. These N-glycans play a crucial role in various biological processes, including protein folding, protein trafficking, and signal transduction [2,3]. The majority of IgG N-glycans possess a conserved core structure characterized by two N-acetylglucosamine (GlcNAc) antennae. These glycans may also exhibit additional modifications, such as core fucose, bisecting GlcNAc, and antennary galactosylation and/or sialylation [4]. IgG N-glycosylation plays a role in various diseases by affecting immune responses and contributing to pathological processes. This is primarily achieved through the interaction of different N-glycan structures attached to the effector domain of IgG with Fcγ receptors and the complement system. The biosynthesis of N-glycans, which are involved in various biological processes, is not solely determined by a direct genetic template. Instead, it is influenced by a combination of multiple genes and environmental factors [5]. This process involves multiple glycosyltransferases and glycosidases, whose expression and activity are regulated by a multitude of factors, including the pH and ion composition of the Golgi apparatus, properties of the Golgi membrane, competition among enzymes, and the abundance of activated sugars [6,7]. These factors serve as the foundation for N-glycan synthesis.

Approximately 50% of plasma glycose variability is heritable, with age and age-related physiological variables accounting for a significant portion of the non-genetic variability [4]. Consequently, the sequence of polysaccharides reflects the integrated effects of individual genetic makeup and the environment [5], as is the case with aging [8]. Glycosylation is a process that relies on the activity of various enzymes, substrate availability, cellular metabolism, and dynamic equilibrium [9]. It is highly sensitive to pathological and physiological conditions, including the process of aging [10,11]. In various inflammatory and autoimmune conditions [12,13,14,15,16], as well as metabolic, cardiovascular, infectious, and neoplastic diseases, there is a notable decrease in sialylation and galactosylation of the IgG Asn297-linked glycan. Consequently, there is an increased prevalence of GlcNAc-terminated glycans. These conditions also have an impact on the presence of core-fucose and bisecting GlcNAc. Significantly, similar glycomic alterations are observed during the aging process [17,18,19,20]. The aberrant glycosylation of IgG glycans associated with inflammation and aging contributes to the sustenance of inflammation through diverse mechanisms, perpetuating a detrimental cycle [17]. These mechanisms encompass complement activation, Fcγ receptor binding, interaction with lectin receptors on antigen-presenting cells, and reactivity with autoantibodies [9]. Currently, many studies have been conducted on the causal relationship between IgG N-glycation and inflammatory and autoimmune diseases [21,22,23]. However, we have limited knowledge about the causal relationship between N-glycosylation of immunoglobulins and aging. The association between aberrant glycosylation of immunoglobulins and the aging process is often unclear, and it is uncertain whether it is a cause or a result of organismal aging.

One of the most rigorous epidemiological designs for studying causal relationships is the randomized controlled trial (RCT). However, conducting an RCT to investigate the relationship between IgG N-glycosylation and aging in humans would pose significant challenges. Mendelian randomization (MR) is a popular approach for exploring potential causal associations between an exposure and an outcome. It leverages genetic variants as instrumental variables (IVs) to mitigate confounding and reverse causation [24,25].

This study aims to investigate the utility of IgG N-glycosylation as a biomarker for aging and explore its potential as a molecular tool to slow down or reverse the aging process. To achieve this, we utilized summary statistics from a previously published genome-wide association study (GWAS) that examined IgG N-glycosylation traits, as well as the frailty index (FI) and leukocyte telomere length (LTL) as indicators of aging. By employing both two-sample Mendelian randomization (MR) and multivariable MR approaches, we sought to estimate the causal impact of IgG N-glycosylation on the aging process. This analysis will provide valuable insights into the relationship between IgG N-glycosylation and aging, shedding light on its potential role as a biomarker and therapeutic target for age-related conditions.

## 2. Results

The characteristics of the harmonized instruments and their associations with the corresponding N-glycosylation traits are presented in Supplementary Table S1.

### 2.1. Two-Sample MR Analysis

**Figure 1:**
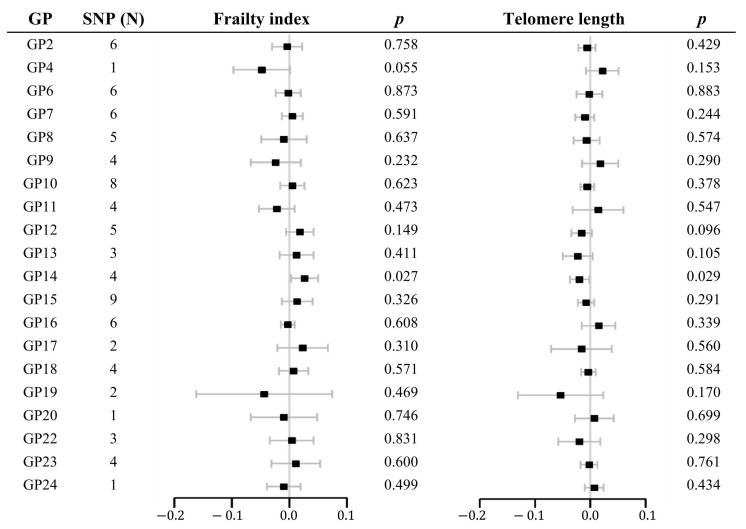
Forest plots of the estimated mendelian randomization effects and 95% confidence intervals for the causal associations of IgG N-glycans with the frailty index or telomere length by standard mendelian randomization analysis using the inverse-variance weighted method. The x-axis indicates β. Dots represent the point estimates of the effect and lines represent 95% confidence intervals. GP, glycan peak; SNP, single-nucleotide polymorphism.

**Figure 2:**
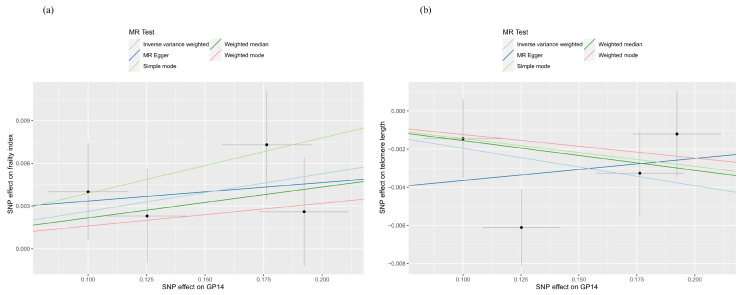
Scatter plots of the causal effect of IgG N-glycosylation on the frailty index and telomere length, with the slope of the line corresponding to the estimated causal effect. (**a**) GP14 on the frailty index; (**b**) GP14 on telomere length.

**Figure 3:**
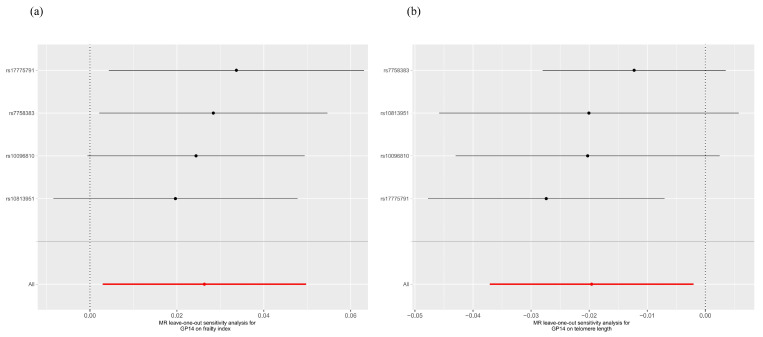
Leave-one-out inverse-variance weighted Mendelian randomization analyses of GP14 on the frailty index and telomere length. (**a**) GP14 on the frailty index; (**b**) GP14 on telomere length.

As shown in Figure 1 and Supplementary Table S2, suggestive evidence of causality was detected in GP14 on FI (β 0.026, 95% CI 0.003 to 0.050, *p* = 0.027) and LTL (β −0.020, 95% CI −0.037 to −0.002, *p* = 0.029). Heterogeneity and pleiotropy were not observed using Cochran’s Q test of the IVW method, MR-Egger intercept, and MR-PRESSO global tests. Scatter and leave-one-out plots of each pair of suggestive associations were also provided for better expression of causality and identification of heterogeneity (Figure 2 and Figure 3).

### 2.2. Multivariable MR Analysis

**Figure 4:**
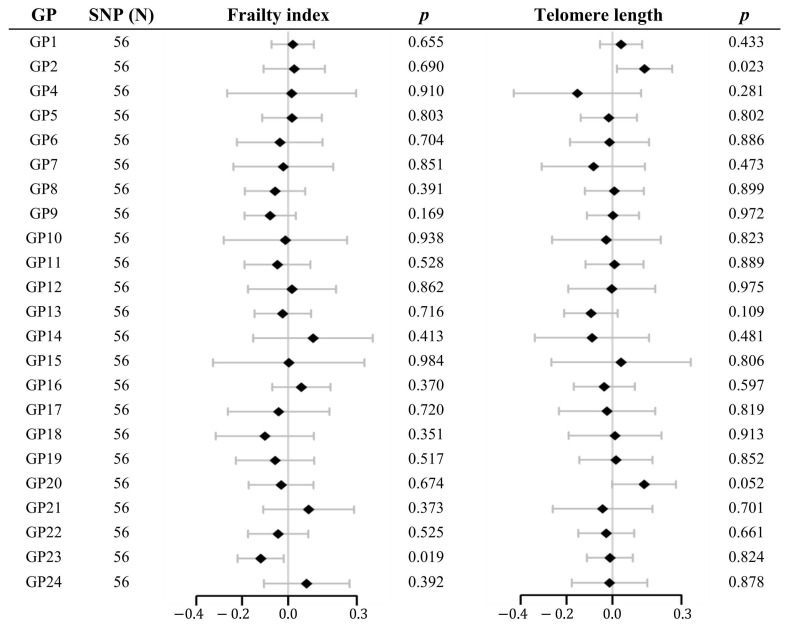
Forest plot of the estimated mendelian randomization effects and 95% confidence intervals for the causal associations of IgG N-glycans with the frailty index and telomere length by multivariable mendelian randomization analysis on the inverse-variance weighted method. The x-axis indicates β. Dots represent the point estimates of the effect and lines represent 95% confidence intervals. GP, glycan peak; SNP, single-nucleotide polymorphism.

As shown in Figure 4 and Supplementary Tables S3 and S4, suggestive evidence of causality was detected in GP23 on FI (β −0.119, 95% CI −0.219 to −0.019, *p* = 0.019) and GP2 on LTL (β 0.140, 95% CI 0.020 to 0.260, *p* = 0.023). Heterogeneity and pleiotropy were not observed using Cochran’s Q test of the IVW method, MR-Egger intercept, or MR-PRESSO global tests.

## 3. Discussion

In this study, the MR method was carried out to investigate the relationship of IgG N-glycosylation with aging. The results of two-sample MR provided evidence of a suggestive causal effect of GP14 on FI and LTL. The results of multivariable MR provided evidence of a suggestive causal effect of GP23 on FI and GP2 on LTL. In this study, we selected the FI and LTL as alternative indicators of aging. A higher FI and shorter LTL signify a more advanced aging state within the body [26,27].

Most studies investigating the impact of aging on IgG glycosylation in adults have consistently observed that in early adulthood, there is a higher prevalence of digalactosylated structures and a lower occurrence of agalactosylated structures in IgG glycosylation patterns [28]. However, as individuals age, there is a noticeable decrease in galactosylation and a concurrent increase in agalactosylation [19,29,30]. The concept of inflammaging suggests that the modulation of inflammation via IgG Fc glycosylation plays a role in the biological aging process. This implies that changes in the glycosylation patterns of IgG Fc region may have an impact on the regulation of inflammation, thus influencing the overall aging trajectory [31]. It proposes that the age-related decline in IgG galactosylation, caused by chronic low-grade sterile inflammation in older individuals, further amplifies inflammation, creating a detrimental cycle in which agalactosylated IgG species serve as both an aging biomarker and a contributor to its pathogenesis [17,32].

Multiple lines of evidence support the role of IgG galactosylation as a modulator of its inflammatory activity. It is suggested that IgG lacking terminal galactoses exhibits pro-inflammatory effects by activating complement through the alternative pathway and by binding to mannose-binding lectin (MBL) to activate the lectin pathway [33,34]. Furthermore, galactosylation of immune complexes is crucial for initiating the anti-inflammatory signaling cascade through binding to the inhibitory receptor FcγRIIB [35]. Conversely, highly galactosylated immune complexes have been shown to inhibit the pro-inflammatory activity of the complement component C5a [36].

Based on our two-sample Mendelian randomization (MR) analysis, we observed a suggestive correlation between the abundance of GP14 (FA2G2) and a higher frailty index (FI), as well as a shorter leukocyte telomere length (LTL), indicating a more advanced aging process. Moreover, the multivariable MR analysis revealed a suggestive association between the abundance of GP2 (A2) and longer LTL, suggesting an inverse relationship with the aging process. GP14 (FA2G2) is a characteristic that describes the proportion of core fucosylated glycans with a digalactosylation structure. GP2 (A2) describes the proportion of agalactosylated IgG N-glycans. However, our results are inconsistent with the observed changes in galactosylation during the aging process. One possible explanation for this discrepancy in two-sample MR may be attributed to the impact of multicollinearity among glycans, which can affect the strength and direction of causal relationships [37]. Furthermore, the nonsignificant association between GP14 and aging observed in subsequent multivariable MR analyses may also confirm this issue. Another potential explanation for this inconsistency is that galactosylation exhibits pro-inflammatory effects, which, in turn, exacerbate the aging process in the body. There are reports indicating a pro-inflammatory role of IgG galactosylation. Terminal galactoses have been found to enhance IgG’s affinity for the C1q complement component, thereby promoting classical pathway complement activation and complement-dependent cytotoxicity (CDC) [38,39]. Additionally, terminal galactoses have been shown to enhance IgG’s affinity for activating Fcγ receptors, leading to enhanced downstream effects, notably, antibody-dependent cell-mediated cytotoxicity (ADCC) [40,41].

Meanwhile, sialylation is observed to change with aging. Maja Pučić Baković et al. [42] reported that the level of sialylated glycoforms significantly decreased with increasing age. Maja Pucić et al. [4] also reached similar conclusions: within IgG glycans, there is a significant decrease in IgG sialylation levels with increasing age, while no age-related variations were observed in the degree of IgG core fucosylation.

In cases of homeostasis disturbance, terminal sialic acids seem to primarily function as regulators, determining the shift between pro-inflammatory and anti-inflammatory activity of IgG. The diminished affinity of highly sialylated IgG for activating FcγRIIIA significantly diminishes both in vivo and in vitro antibody-dependent cell-mediated cytotoxicity (ADCC) by natural killer (NK) cells and enhances anti-inflammatory responses [43,44,45]. The interaction between sialylated IgG and lectin receptors, such as dendritic cell-specific intercellular adhesion molecule grabbing non-integrin, C-type lectin domain family 4 member A, and B-cell receptor CD22, is considered to contribute to the resolution of inflammation by triggering the release of T helper 2 cytokines and, subsequently, elevating the activation threshold of adaptive and innate immune cells [46]. In addition, based on mouse experiments, a proposed model suggests that upon binding of α2,6-sialylated antibodies to DC-SIGN, dendritic cells release IL-33, which, in turn, triggers the expansion of IL-4-producing basophils [47]. These two Th2 cytokines subsequently upregulate the inhibitory FcγRIIb receptor in macrophages, leading to the suppression of inflammation [48].

By conducting multivariable MR analysis and adjusting for other IgG N-glycans, we obtained a suggestive correlation between the abundance of GP23 (FA2G2S2) and a lower frailty index (FI), indicating a more youthful aging process. GP23 (FA2G2S2) is a characteristic that describes the proportion of core fucosylated glycans with a disialylation structure. Our research findings are in line with the notion that the sialylation of IgG contributes to an effective reduction in the body’s inflammation levels, thus leading to a deceleration of the aging process [49].

Our study exhibits several notable strengths that contribute to its robustness and validity. One key strength lies in the implementation of a two-sample Mendelian randomization (MR) analysis and a multivariable MR analysis. These analytical approaches utilized genetic variants as instrumental variables to estimate the relationship between IgG N-glycosylation levels and aging, capitalizing on large-scale genome-wide association study (GWAS) databases. By employing these methods, we effectively addressed concerns related to reverse causation and residual confounding, enhancing the reliability of our findings. Furthermore, we conducted a comprehensive set of sensitivity analyses to assess the robustness of our results. Finally, to minimize potential biases stemming from population stratification, we exclusively focused on individuals of European ancestry. This deliberate selection criterion reduced the influence of population-specific factors and helped ensure the generalizability of our findings within the European population.

This study has several limitations that should be considered. First, the generalizability of our findings may be limited to individuals of European ancestry, as our data exclusively included participants from this population. Therefore, caution should be exercised when extrapolating these results to Asian and African populations. Second, we encountered challenges in estimating the impact of IgG N-glycan traits on aging due to the relatively lower statistical power of the IgG N-glycome genome-wide association studies (GWAS) compared to GWAS studies on the diseases themselves. As a result, we were only able to incorporate a maximum of nine instrumental variables (IVs) per glycan trait, which might have an impact on the precision of our estimates. Third, the absence of individual-level data restricted our ability to perform stratified analyses or adjust for additional covariates beyond those provided in the summary statistics dataset. This limitation should be kept in mind when interpreting our findings. Fourth, the ultraperformance liquid chromatography (UPLC) method for quantifying IgG glycans does not permit the discrimination between glycans linked to the Fc and Fab domains. Given that glycosylation occurs in only 15%–20% of the Fab segment of IgG, the identified associations are more likely attributed to discrepancies in Fc glycans [50]. However, it is important to acknowledge that the potential influence of modifications in Fab N-glycosylation cannot be entirely discounted. Last, despite our efforts to minimize confounding and ensure robustness through various methods, residual confounders may still exist in our analyses. It is important to acknowledge that even with rigorous techniques, the presence of unmeasured or unknown confounding factors cannot be completely ruled out. Furthermore, we refrained from employing the Bonferroni correction method on the *p*-values, thus underscoring that the results obtained merely serve as suggestive rather than conclusive evidence of causal relationships.

## 4. Materials and Methods

### 4.1. Study Design

In our current study, we employed a two-sample Mendelian randomization (MR) approach, which does not require genetic associations for both analysed traits to be available within the same cohort of individuals. Instead, it is only necessary to have selected genetic variants or reliable linkage disequilibrium (LD) proxies present in both cohorts, enabling us to conduct the analysis effectively. Previous studies have documented robust intra-associations among IgG N-glycans [4], indicating mutual influences between different IgG N-glycosylation traits. These associations may arise from shared regulatory mechanisms [37]. To delve deeper into the relationship between exposure (each IgG N-glycan) and outcome, we employed a multivariable MR analysis [51]. To comprehensively explore the potential associations, we aggregated all IgG N-glycans as instrumental variables (IVs) for each individual IgG N-glycan which enabled us to thoroughly examine the relationships between IgG N-glycosylation and the variables of interest [22].

### 4.2. Data Source

We used genetic variants robustly associated with glycosylation of IgG identified in a meta-analysis of a genome-wide association study (GWAS) for the four cohorts CROATIA-Korcula, CROATIA-Vis, ORCADES, and TwinsUK (*n* total = 8090) from the European population [52]. The GWAS summarized data can be downloaded at https://doi.org/10.7488/ds/2481 (accessed on 15 December 2022).

The genetic variants significantly associated with FI were obtained through a GWAS meta-analysis conducted by Atkins et al. [53] using data from 164,610 UK Biobank participants (79,791 males and 84,819 females, with an average age of 64.1 ± 2.8 years) and 10,616 TwinGene participants (5039 males and 5577 females, with an average age of 58.3 ± 7.9 years). The Rockwood frailty index utilized a deficit accumulation model. Each individual was assigned a score of 0 or 1 based on their compliance with each deficit (0 indicating non-compliance). The FI for each participant was subsequently calculated by dividing the number of deficits by the total of 49 deficits outlined in the previous study. This approach provided a quantitative measure of frailty based on the accumulation of deficits.

Data sources for LTL were taken from the UK Biobank, including 472,174 UKB participants [54]. Leukocyte telomere length was quantified by measuring the ratio of telomere repeat copy number (T) to that of a single copy gene (S) in DNA extracted from peripheral blood leukocytes. The measurements were obtained at baseline using a multiplex quantitative polymerase chain reaction (PCR) method. To account for technical factors, the LTL measurements (T/S ratios) were adjusted and log and z-transformations were applied to the included samples prior to analysis. This preprocessing step ensured standardized and normalized LTL measurements for further statistical examination.

The datasets used for both the exposure (IgG N-glycosylation) and outcome (aging proxy indicators) were primarily derived from individuals of European ancestry or datasets specifically focused on European populations (Table 1). Importantly, there was minimal to no significant crossover or overlap between the participants included in the genome-wide association studies (GWAS) conducted for IgG N-glycosylation and the GWASs conducted for aging proxy indicators. This ensures the independence and integrity of the datasets, allowing for robust analyses of the associations between IgG N-glycosylation and aging-related factors.

### 4.3. Isolation of IgG and Glycan Analysis

The process of isolating IgG and conducting glycan analysis has been thoroughly documented in previous studies [37,52,55]. In summary, IgG isolation involved employing affinity chromatography, where binding to protein G plates was utilized. Subsequently, glycans were released and labeled with 2-AB (2-aminobenzamide) fluorescent dye. Hydrophilic interaction ultraperformance liquid chromatography (UPLC) was then employed to separate and quantify the glycans, resulting in 24 distinct chromatographic peaks. The GP1, 2, 4–24 traits corresponded to peaks observed in UPLC analysis and represented the approximate proportion of a specific glycan (the most prevalent glycan within the peak) in relation to the total IgG glycan composition. These traits provide insights into the levels of galactosylation, sialylation, and core fucosylation [4].

### 4.4. Instrumental Variable Selection Criteria

SNPs associated with IgG N-glycosylation traits that reached genome-wide significance (*p* < 5 × 10^−8^) were selected as instrumental variables (IVs). We selected independent genetic variants using the cut-off of linkage disequilibrium (LD) value (threshold set at r^2^ = 0.001) to ensure that the IVs for N-glycosylation traits were independent.

To establish causal relationships, we utilized single nucleotide polymorphisms (SNPs) that demonstrated significant genome-wide associations with IgG N-glycosylation traits (*p* < 5 × 10^−8^) as instrumental variables (IVs). To ensure the independence of the IVs for N-glycosylation traits, we applied a specific threshold set at r^2^ = 0.001 based on linkage disequilibrium (LD) values. This criterion allowed us to select genetic variants that were not strongly correlated with each other, thus ensuring their independence in our analysis [56]. The F-statistic was calculated for each selected IV to evaluate its instrument strength. If the value was below 10, it was considered a weak IV [57].

### 4.5. Statistical Analysis

We employed the multiplicative random-effects inverse-variance weighted (IVW) model as our primary statistical approach (for analysis with ≥2 SNPs) [58]. To assess the heterogeneity of instrumental variables (IVs), we conducted the Cochran Q statistic. In cases where significant heterogeneity was detected, we employed the random-effects IVW method. Conversely, when heterogeneity was not observed, we utilized the fixed-effects IVW method [59].

To address potential violations of valid instrumental variable (IV) assumptions, we performed sensitivity analyses using various methods. These included the simple mode, weighted mode, weighted median, MR-Egger, and Mendelian randomization pleiotropy RESidual sum and outlier (MR-PRESSO). To identify any potential horizontal pleiotropic outliers in our multi-instrument summary-level MR testing, we employed the MR-PRESSO method [60]. To assess deviations from the standard instrumental variable assumptions, we utilized MR-Egger regression. This method provided an effect estimate that remained unaffected by any potential deviations, ensuring the robustness of our results. Moreover, if the *p*-value of the MR-Egger intercept exceeded 0.05, it indicated the absence of any horizontal pleiotropic effects [61]. Conducting a leave-one-out analysis, we systematically excluded individual variants from the analysis to assess their impact on the overall MR estimate. This analysis allowed us to evaluate the influence of specific genetic variants on our results and identify any potential sources of bias or distortion [62]. Furthermore, scatter plots were plotted for sensitivity analyses.

The data analyses were performed with the “TwoSampleMR”, “MR-PRESSO”, and “MendelianRandomization” packages. Data cleaning and statistical analysis were performed using R version 4.2.2 (https://www.r-project.org/ (accessed on 1 September 2022)). For two-sample MR and multivariable MR, a *p* value < 0.05 was considered evidence of a potential association.

## 5. Conclusions

In conclusion, our research findings provide support for a potential causal relationship between lower GP23 (FA2G2S2) and a more advanced state of aging in the body. Further conducting of larger-scale GWASs, identifying novel genetic variants associated with IgG N-glycosylation, having a sufficient number of instrumental variables (IVs), and selecting more appropriate aging indicators, are crucial for validating the causal relationship between IgG N-glycosylation and aging. In the future, if possible, we plan to conduct functional studies to validate the existence of causal associations based on the results of the genetic association study.

## Figures and Tables

**Table 1:** Overview of the summary data.

Characteristic	Resource	Sample Size	Population Ancestry	Data Download
Exposure				
IgG N-glycosylation	CROATIA-Korcula (recruited from the town of Korčula and the villages of Lumbarda, Žrnovo and Račišće.), CROATIA-Vis (recruited from the villages of Vis and Komiža on the Dalmatian island of Vis), ORCADES (recruited from the isolated Scottish archipelago of Orkney), and TwinsUK (recruited from the United Kingdom female adult twin)	8090	European	https://doi.org/10.7488/ds/2481 (accessed on 15 December 2022)
Outcome				
Frailty index	UK Biobank (recruited from the England, Scotland and Wales), and TwinGene (recruited from the Sweden adult twin)	175,226	European	https://doi.org/10.6084/m9.figshare.9204998.v4 (accessed on 15 December 2022)
Telomere length	UK Biobank	472,174	European	https://figshare.com/s/caa99dc0f76d62990195 (accessed on 15 December 2022)

## Data Availability

The original contributions presented in the study are included in the article. Further inquiries can be directed to the corresponding authors.

## Supplementary Materials

The following supporting information can be downloaded at: https://www.mdpi.com/article/10.3390/molecules29061281/s1, Table S1. Characteristics of SNPs associated with IgG N-glycosylation traits; Table S2. Estimates of the causal effect of IgG N-glycosylation on the frailty index and telomere length via two-sample MR; Table S3. Estimates of the causal effect of IgG N-glycosylation on the frailty index via multivariable MR; Table S4. Estimates of the causal effect of IgG N-glycosylation on telomere length via multivariable MR.
