# Supplementary material for: The Causality between Human Immunoglobulin G (IgG) N-Glycosylation and Aging: A Mendelian Randomization Study
Source: Molecules. 2024 Mar 14;29(6):1281. doi: 10.3390/molecules29061281 (PMC10975104; doi:10.3390/molecules29061281)
Supplement: Supplementary file 1 [file molecules-29-01281-s001.zip › molecules-2897268-supplementary.pdf]

SUPPLEMENTAL MATERIAL

The Causality between Human Immunoglobulin G (IgG) N-Glycosylation and Aging: A Mendelian Randomization Study

Wenxin Sun [1], Xuening Jian [1], Jie Zhang [1], Xiaoni Meng [1], Haotian Wang [1], Deqiang Zheng [1], Lijuan Wu [1] and Youxin Wang [2,3,4,*]

1 School of Public Health, Capital Medical University, Beijing 100069, China; sunwx_ada@163.com (W.S.) ; jian_0628@126.com (X.J.); zhangjie@ccmu.edu.cn (J.Z.); mengxiaoni385@163.com (X.M.); sdwanghaotian@126.com (H.W.); deqiangzheng@163.com (D.Z.); wujuan811017@163.com (L.W.)

2 Beijing Key Laboratory of Clinical Epidemiology, Capital Medical University, Beijing 100069, China

3 School of Public Health, North China University of Science and Technology, Tangshan 063210, China

4 Centre for Precision Medicine, Edith Cowan University, Perth 6027, Australia

* Correspondence: wangy@ccmu.edu.cn or sdwangyouxin@163.com

Contents

Supplementary Table S1. Characteristics of SNPs associated with IgG N-glycosylation traits
Supplementary Table S2. Estimates of the causal effect of IgG N-glycosylation on frailty index and telomere length via two-sample MR
Supplementary Table S3. Estimates of the causal effect of IgG N-glycosylation on the frailty index via multivariable MR
Supplementary Table S4. Estimates of the causal effect of IgG N-glycosylation on telomere length via multivariable MR

Supplementary Table S1. Characteristics of SNPs associated with IgG N-glycosylation traits

SNPs	EA	RA	EAF	N	Beta	SE	*p*	F
**GP2**								
rs10483775	A	G	0.017	7949	-0.436	0.065	1.50E-11	44.99
rs11748193	A	T	0.399	7648	0.110	0.018	4.31E-10	37.35
rs11847263	T	G	0.647	8044	-0.273	0.018	1.32E-54	230.03
rs7789913	T	C	0.395	8089	-0.154	0.017	1.36E-19	82.06
rs7812088	A	G	0.119	8089	-0.250	0.026	2.06E-22	92.46
rs907091	T	C	0.490	7980	-0.133	0.017	1.23E-14	61.21
**GP4**								
rs10813951	A	G	0.732	7943	-0.151	0.019	1.27E-15	63.16
**GP6**								
rs17630758	A	G	0.152	8041	-0.161	0.023	1.66E-12	49.00
rs3824459	T	C	0.015	8008	-0.447	0.075	2.79E-09	35.52
rs6421315	C	G	0.383	7659	-0.105	0.018	3.59E-09	34.03
rs7281587	A	G	0.247	8090	-0.142	0.019	2.18E-13	55.86
rs7812088	A	G	0.119	8089	-0.196	0.026	2.20E-14	56.83
rs909674	A	C	0.734	8090	-0.173	0.019	2.59E-19	82.91
**GP7**								
rs11557466	T	C	0.473	8034	0.134	0.017	7.79E-15	62.13
rs11651000	A	G	0.150	8034	0.154	0.024	6.14E-11	41.17
rs11847263	T	G	0.647	8044	-0.263	0.018	4.91E-51	213.48
rs12587616	T	G	0.017	7949	-0.373	0.066	1.25E-08	31.94
rs7789913	T	C	0.395	8089	-0.162	0.017	2.13E-21	90.81
rs7812088	A	G	0.119	8089	-0.198	0.026	1.30E-14	57.99
**GP8**								
rs17303508	T	C	0.612	8054	-0.094	0.017	3.53E-08	30.57
rs2534669	A	G	0.137	7785	-0.159	0.025	2.24E-10	40.45
rs2834838	A	G	0.917	7900	-0.168	0.031	4.59E-08	29.37
rs4074453	T	C	0.758	8089	0.133	0.019	5.90E-12	49.00
rs7865745	A	G	0.318	8053	0.103	0.018	9.03E-09	32.74
**GP9**								
rs10813950	A	G	0.319	8077	0.120	0.018	2.85E-11	44.44
rs2395488	A	G	0.651	8090	0.116	0.019	7.63E-10	37.27
rs4074453	T	C	0.758	8089	-0.215	0.019	1.52E-28	128.05
rs7621161	A	C	0.284	7656	0.152	0.019	3.89E-16	64.00
**GP10**								
rs17630758	A	G	0.152	8041	-0.295	0.023	5.23E-38	164.51
rs3848370	C	G	0.224	3611	0.183	0.034	4.93E-08	28.97
rs4561508	T	C	0.105	8002	0.163	0.028	5.70E-09	33.89
rs4821897	A	G	0.260	7860	0.215	0.020	1.47E-27	115.56
rs6570330	A	G	0.427	8090	-0.140	0.017	4.35E-16	67.82
rs7257072	T	C	0.515	8090	-0.122	0.017	1.59E-13	51.50

rs7789913	T	C	0.395	8089	-0.131	0.017	1.89E-14	59.38
rs8080583	A	C	0.216	6063	0.144	0.025	6.26E-09	33.18
rs9640023	A	G	0.221	7736	-0.117	0.021	1.21E-08	31.04
**GP11**								
rs17630758	A	G	0.152	8041	-0.234	0.023	9.45E-25	103.51
rs4821897	A	G	0.260	7860	0.151	0.020	1.61E-14	57.00
rs7747738	T	C	0.031	5898	0.338	0.062	4.31E-08	29.72
rs9385856	T	C	0.574	8089	0.106	0.017	3.84E-10	38.88
**GP12**								
rs11557467	T	G	0.510	8090	0.101	0.017	4.66E-09	35.30
rs11650354	T	C	0.167	8090	0.138	0.023	9.15E-10	36.00
rs11847263	T	G	0.647	8044	-0.234	0.018	1.43E-40	169.00
rs1555926	T	C	0.789	8090	-0.118	0.020	6.96E-09	34.81
rs6421315	C	G	0.383	7659	-0.120	0.018	2.15E-11	44.44
**GP13**								
rs17775791	T	C	0.739	7759	-0.168	0.019	1.31E-18	78.18
rs7789913	T	C	0.395	8089	-0.123	0.017	5.40E-13	52.35
rs874232	T	C	0.565	8090	0.119	0.017	7.85E-13	49.00
**GP14**								
rs10096810	A	G	0.627	8090	0.100	0.017	4.47E-09	34.60
rs10813951	A	G	0.732	7943	0.176	0.019	1.61E-20	85.81
rs17775791	T	C	0.740	7759	-0.192	0.019	4.81E-24	102.12
rs7758383	A	G	0.521	8020	0.125	0.017	9.61E-14	54.07
**GP15**								
rs17630758	A	G	0.152	8041	-0.181	0.023	2.23E-15	61.93
rs17775791	T	C	0.740	7759	-0.155	0.019	4.14E-16	66.55
rs199456	T	C	0.196	8064	0.162	0.022	6.76E-14	54.22
rs4821897	A	G	0.260	7860	0.140	0.020	1.15E-12	49.00
rs6421315	C	G	0.383	7659	-0.102	0.018	1.17E-08	32.11
rs6570330	A	G	0.427	8090	-0.125	0.017	4.06E-13	54.07
rs6964421	T	C	0.328	8090	0.117	0.018	5.31E-11	42.25
rs741706	T	C	0.543	7648	-0.105	0.017	6.15E-10	38.15
rs9319617	T	C	0.499	7750	0.103	0.017	1.26E-09	36.71
**GP16**								
rs1122979	A	G	0.112	8029	0.202	0.027	6.33E-14	55.97
rs2745851	A	G	0.377	8087	0.095	0.017	3.95E-08	31.23
rs3099844	A	C	0.136	8090	-0.227	0.031	1.12E-13	53.62
rs3777178	C	G	0.215	8015	-0.140	0.020	3.80E-12	49.00
rs4074453	T	C	0.758	8089	-0.197	0.019	1.33E-24	107.50
rs6764279	A	C	0.285	8086	-0.498	0.018	7.20E-167	765.44
**GP17**								
rs11847263	T	G	0.647	8044	-0.191	0.017	4.33E-28	126.23
rs6583437	A	G	0.372	7788	-0.095	0.017	4.22E-08	31.23
**GP18**								

rs10813951	A	G	0.732	7943	0.230	0.019	8.84E-34	146.54
rs250555	T	C	0.860	7647	-0.155	0.025	6.83E-10	38.44
rs2725391	T	C	0.460	8090	0.100	0.017	1.95E-09	34.60
rs7621161	A	C	0.284	7656	-0.193	0.019	2.37E-25	103.18
**GP19**								
rs4424066	A	G	0.590	8090	0.111	0.018	7.45E-10	38.03
rs8137426	T	G	0.255	7763	0.123	0.020	4.63E-10	37.82
**GP20**								
rs12986368	T	C	0.371	7717	0.116	0.018	3.39E-11	41.53
**GP22**								
rs3742597	A	G	0.750	8090	-0.153	0.019	1.03E-15	64.84
rs6583437	A	G	0.372	7788	-0.125	0.017	5.56E-13	54.07
rs7621161	A	C	0.284	7656	-0.110	0.018	2.06E-09	37.35
**GP23**								
rs10813951	A	G	0.732	7943	0.176	0.019	1.37E-20	85.81
rs4649042	A	G	0.360	8019	-0.101	0.017	6.66E-09	35.30
rs7621161	A	C	0.284	7656	-0.179	0.019	4.28E-22	88.76
rs909674	A	C	0.734	8090	0.186	0.019	2.16E-22	95.83
**GP24**								
rs7621161	A	C	0.284	7656	-0.252	0.019	1.65E-42	175.91

IgG, immunoglobulin G; GP, glycan peak; SNP, single-nucleotide polymorphism; EA, effect allele; OA, other allele; SE, standard error; MAF, minor allele frequency.

Supplementary Table S2. Estimates of the causal effect of IgG N-glycosylation on frailty index and telomere length via two-sample MR

Exposure	Outcome	Method	Ivs	β(95%CI)	*p*
GP2	Frailty index	Inverse variance weighted	6	-0.004(-0.030,0.022)	0.758
		Q-statistic			0.022
		Simple mode	6	-0.020(-0.058,0.019)	0.362
		Weighted mode	6	0.021(-0.004,0.046)	0.153
		Weighted median	6	-0.001(-0.025,0.023)	0.933
		MR Egger	6	0.041(-0.020,0.101)	0.259
		egger_intercept			0.193
		MR-PRESSO	6	0.008(-0.010,0.026)	0.423
		global test			0.168
	Telomere length	Inverse variance weighted	6	-0.006(-0.021,0.009)	0.429
		Q-statistic			0.030
		Simple mode	6	-0.001(-0.022,0.020)	0.940
		Weighted mode	6	0.001(-0.013,0.015)	0.900
		Weighted median	6	-0.002(-0.015,0.011)	0.790
		MR Egger	6	0.024(-0.006,0.054)	0.198
		egger_intercept			0.104
		MR-PRESSO	6	-0.007(-0.018,0.004)	0.288
		global test			0.233
GP4	Frailty index	Wald ratio	1	-0.048(-0.097,0.001)	0.055
	Telomere length	Wald ratio	1	0.022(-0.008,0.051)	0.153
GP6	Frailty index	Inverse variance weighted	6	-0.002(-0.023,0.020)	0.873
		Q-statistic			0.852
		Simple mode	6	-0.004(-0.045,0.036)	0.841
		Weighted mode	6	-0.005(-0.045,0.035)	0.832
		Weighted median	6	-0.004(-0.031,0.023)	0.787
		MR Egger	6	-0.015(-0.083,0.053)	0.694
		egger_intercept			0.714
		MR-PRESSO	6	-0.002(-0.011,0.008)	0.773
		global test			0.910
	Telomere length	Inverse variance weighted	6	-0.002(-0.025,0.022)	0.883
		Q-statistic			0.007
		Simple mode	6	0.014(-0.01,0.039)	0.305
		Weighted mode	6	0.01(-0.015,0.035)	0.476
		Weighted median	6	0.005(-0.012,0.022)	0.552
		MR Egger	6	0.062(0.015,0.108)	0.061
		egger_intercept			0.048
		MR-PRESSO	6	0.003(-0.014,0.021)	0.723
		global test			0.081
GP7	Frailty index	Inverse variance weighted	6	0.005(-0.013,0.023)	0.591
		Q-statistic			0.327

		Simple mode	6	-0.020(-0.063,0.023)	0.400
		Weighted mode	6	0.021(-0.004,0.045)	0.165
		Weighted median	6	0.012(-0.011,0.035)	0.313
		MR Egger	6	0.027(-0.038,0.093)	0.461
		egger_intercept			0.524
		MR-PRESSO	6	-0.004(-0.019,0.011)	0.641
		global test			0.448
		Inverse variance weighted	6	-0.010(-0.028,0.007)	0.244
		Q-statistic			0.013
		Simple mode	6	-0.031(-0.067,0.004)	0.147
		Weighted mode	6	0.000(-0.017,0.018)	0.976
	Telomere length	Weighted median	6	-0.007(-0.021,0.007)	0.327
		MR Egger	6	0.038(-0.003,0.080)	0.147
		egger_intercept			0.075
		MR-PRESSO	6	-0.013(-0.029,0.002)	0.155
		global test			0.106
		Inverse variance weighted	5	-0.010(-0.049,0.030)	0.637
		Q-statistic			0.103
		Simple mode	5	0.004(-0.041,0.050)	0.860
		Weighted mode	5	0.008(-0.035,0.051)	0.741
	Frailty index	Weighted median	5	0.003(-0.033,0.039)	0.868
		MR Egger	5	0.096(-0.077,0.269)	0.356
		egger_intercept			0.307
		MR-PRESSO	5	0.010(-0.038,0.058)	0.717
		global test			0.100
GP8		Inverse variance weighted	5	-0.007(-0.031,0.017)	0.574
		Q-statistic			0.102
		Simple mode	5	-0.022(-0.054,0.010)	0.249
		Weighted mode	5	-0.023(-0.050,0.005)	0.183
	Telomere length	Weighted median	5	-0.019(-0.041,0.004)	0.105
		MR Egger	5	-0.107(-0.186,-0.029)	0.075
		egger_intercept			0.083
		MR-PRESSO	5	0.011(-0.009,0.031)	0.328
		global test			0.237
		Inverse variance weighted	4	-0.024(-0.067,0.020)	0.282
		Q-statistic			0.017
		Simple mode	4	-0.071(-0.147,0.004)	0.160
		Weighted mode	4	-0.002(-0.032,0.029)	0.928
	Frailty index	Weighted median	4	-0.010(-0.042,0.022)	0.525
GP9		MR Egger	4	0.087(-0.054,0.227)	0.350
		egger_intercept			0.252
		MR-PRESSO	4	0.010(-0.038,0.058)	0.717
		global test			0.100
	Telomere length	Inverse variance weighted	4	0.018(-0.015,0.050)	0.290

		Q-statistic			0.001
		Simple mode	4	0.010(-0.012,0.032)	0.441
		Weighted mode	4	0.011(-0.007,0.029)	0.314
		Weighted median	4	0.013(-0.005,0.031)	0.164
		MR Egger	4	-0.034(-0.175,0.108)	0.686
		egger_intercept			0.539
		MR-PRESSO(Outlier-corrected)	4	0.008(0.002,0.015)	0.235
		global test			0.014
		Inverse variance weighted	9	0.005(-0.016,0.026)	0.660
		Q-statistic			0.059
		Simple mode	9	0.011(-0.015,0.037)	0.437
		Weighted mode	9	0.009(-0.013,0.030)	0.469
	Frailty index	Weighted median	9	0.006(-0.014,0.027)	0.533
		MR Egger	9	0.023(-0.054,0.100)	0.574
		egger_intercept			0.639
		MR-PRESSO	9	0.009(-0.010,0.029)	0.364
		global test			0.103
GP10		Inverse variance weighted	9	-0.006(-0.018,0.007)	0.378
		Q-statistic			0.051
		Simple mode	9	-0.005(-0.021,0.011)	0.556
		Weighted mode	9	-0.006(-0.020,0.008)	0.415
	Telomere length	Weighted median	9	-0.007(-0.019,0.005)	0.282
		MR Egger	9	0.001(-0.045,0.047)	0.969
		egger_intercept			0.776
		MR-PRESSO	9	-0.002(-0.014,0.01)	0.702
		global test			0.069
		Inverse variance weighted	4	-0.011(-0.042,0.020)	0.473
		Q-statistic			0.155
		Simple mode	4	0.000(-0.046,0.047)	0.990
		Weighted mode	4	-0.001(-0.040,0.037)	0.957
	Frailty index	Weighted median	4	-0.003(-0.033,0.026)	0.826
		MR Egger	4	-0.055(-0.128,0.018)	0.279
		egger_intercept			0.333
		MR-PRESSO	4	-0.001(-0.024,0.022)	0.945
		global test			0.367
GP11		Inverse variance weighted	4	0.014(-0.032,0.060)	0.547
		Q-statistic			<0.001
		Simple mode	4	-0.010(-0.030,0.010)	0.385
		Weighted mode	4	-0.010(-0.028,0.008)	0.346
	Telomere length	Weighted median	4	-0.010(-0.028,0.008)	0.272
		MR Egger	4	0.076(-0.037,0.189)	0.320
		egger_intercept			0.367
		MR-PRESSO(Outlier-corrected)	4	-0.010(-0.015,-0.005)	0.169
		global test			0.007

GP12	Frailty index	Inverse variance weighted	5	0.018(-0.006,0.041)	0.149
		Q-statistic			0.287
		Simple mode	5	0.015(-0.024,0.055)	0.486
		Weighted mode	5	0.026(-0.003,0.054)	0.154
		Weighted median	5	0.022(-0.004,0.048)	0.093
		MR Egger	5	0.060(-0.006,0.126)	0.175
		egger_intercept			0.278
		MR-PRESSO	5	-0.011(-0.041,0.018)	0.489
		global test			0.251
	Telomere length	Inverse variance weighted	5	-0.016(-0.035,0.003)	0.096
		Q-statistic			0.075
		Simple mode	5	-0.017(-0.051,0.017)	0.381
		Weighted mode	5	-0.005(-0.021,0.012)	0.618
		Weighted median	5	-0.006(-0.023,0.010)	0.454
		MR Egger	5	0.023(-0.022,0.069)	0.384
		egger_intercept			0.168
		MR-PRESSO	5	-0.014(-0.033,0.006)	0.239
		global test			0.149
GP13	Frailty index	Inverse variance weighted	3	0.012(-0.017,0.042)	0.411
		Q-statistic			0.914
		Simple mode	3	0.017(-0.023,0.057)	0.501
		Weighted mode	3	0.016(-0.022,0.055)	0.495
		Weighted median	3	0.015(-0.018,0.049)	0.375
		MR Egger	3	0.027(-0.160,0.215)	0.823
		egger_intercept			0.899
		MR-PRESSO	3	-	-
		global test			-
	Telomere length	Inverse variance weighted	3	-0.023(-0.050,0.005)	0.105
		Q-statistic			0.088
		Simple mode	3	-0.011(-0.040,0.018)	0.537
		Weighted mode	3	-0.010(-0.035,0.015)	0.518
		Weighted median	3	-0.013(-0.035,0.009)	0.258
		MR Egger	3	0.056(-0.135,0.248)	0.667
		egger_intercept			0.563
		MR-PRESSO	3	-	-
		global test			-
GP14	Frailty index	Inverse variance weighted	4	0.026(0.003,0.050)	0.027
		Q-statistic			0.762
		Simple mode	4	0.039(-0.002,0.080)	0.157
		Weighted mode	4	0.016(-0.021,0.053)	0.458
		Weighted median	4	0.022(-0.007,0.050)	0.139
		MR Egger	4	0.013(-0.081,0.107)	0.812
		egger_intercept			0.801
		MR-PRESSO	4	0.801(0.797,0.805)	0.016

		global test			0.240
		Inverse variance weighted	4	-0.020(-0.037,-0.002)	0.029
		Q-statistic			0.200
		Simple mode	4	-0.015(-0.040,0.011)	0.344
		Weighted mode	4	-0.012(-0.033,0.008)	0.321
	Telomere length	Weighted median	4	-0.016(-0.033,0.002)	0.085
		MR Egger	4	0.012(-0.063,0.086)	0.789
		egger_intercept			0.485
		MR-PRESSO	4	-0.018(-0.036,-0.001)	0.133
		global test			0.313
		Inverse variance weighted	9	0.013(-0.013,0.040)	0.326
		Q-statistic			0.030
		Simple mode	9	0.009(-0.025,0.043)	0.612
		Weighted mode	9	0.010(-0.022,0.043)	0.551
	Frailty index	Weighted median	9	0.012(-0.014,0.037)	0.364
		MR Egger	9	0.055(-0.086,0.197)	0.469
		egger_intercept			0.571
		MR-PRESSO(Outlier-corrected)	9	0.003(-0.019,0.026)	0.777
GP15		global test			0.031
		Inverse variance weighted	9	-0.008(-0.023,0.007)	0.291
		Q-statistic			0.071
		Simple mode	9	-0.011(-0.031,0.010)	0.332
		Weighted mode	9	-0.010(-0.030,0.010)	0.338
	Telomere length	Weighted median	9	-0.009(-0.024,0.005)	0.212
		MR Egger	9	0.035(-0.040,0.109)	0.396
		egger_intercept			0.293
		MR-PRESSO(Outlier-corrected)	9	0.004(-0.007,0.014)	0.501
		global test			0.041
		Inverse variance weighted	6	-0.003(-0.015,0.009)	0.623
		Q-statistic			0.782
		Simple mode	6	-0.006(-0.029,0.016)	0.605
		Weighted mode	6	-0.006(-0.019,0.008)	0.450
	Frailty index	Weighted median	6	-0.005(-0.019,0.008)	0.431
		MR Egger	6	-0.011(-0.034,0.012)	0.473
		egger_intercept			0.714
GP16		MR-PRESSO(Outlier-corrected)	6	-0.006(-0.015,0.003)	0.277
		global test			0.880
		Inverse variance weighted	6	0.015(-0.016,0.045)	0.339
		Q-statistic			<0.001
		Simple mode	6	0.005(-0.009,0.020)	0.500
	Telomere length	Weighted mode	6	0.004(-0.004,0.012)	0.386
		Weighted median	6	0.004(-0.005,0.012)	0.382
		MR Egger	6	-0.004(-0.064,0.057)	0.912
		egger_intercept			0.520

		MR-PRESSO(Outlier-corrected)	6	0.007(-0.005,0.019)	0.330
		global test			0.003
GP17	Frailty index	Inverse variance weighted	2	0.023(-0.021,0.067)	0.309
		Q-statistic			0.166
	Telomere length	Inverse variance weighted	2	-0.016(-0.071,0.039)	0.560
		Q-statistic			0.004
GP18	Frailty index	Inverse variance weighted	4	0.007(-0.018,0.033)	0.571
		Q-statistic			0.237
		Simple mode	4	-0.012(-0.059,0.034)	0.640
		Weighted mode	4	-0.013(-0.055,0.028)	0.580
		Weighted median	4	0.004(-0.024,0.033)	0.761
		MR Egger	4	0.035(-0.062,0.132)	0.556
		egger_intercept			0.621
		MR-PRESSO	4	-0.006(-0.033,0.02)	0.668
		global test			0.293
	Telomere length	Inverse variance weighted	4	-0.004(-0.017,0.009)	0.584
		Q-statistic			0.369
		Simple mode	4	0.008(-0.021,0.036)	0.636
		Weighted mode	4	-0.013(-0.036,0.009)	0.329
		Weighted median	4	0.000(-0.016,0.017)	0.988
		MR Egger	4	-0.004(-0.059,0.051)	0.903
		egger_intercept			0.995
		MR-PRESSO(Outlier-corrected)	4	0.003(-0.009,0.016)	0.628
		global test			0.425
GP19	Frailty index	Inverse variance weighted	2	-0.044(-0.162,0.074)	0.469
		Q-statistic			0.005
	Telomere length	Inverse variance weighted	2	-0.054(-0.131,0.023)	0.170
		Q-statistic			0.003
GP20	Frailty index	Wald ratio	1	-0.010(-0.067,0.048)	0.746
	Telomere length	Wald ratio	1	0.007(-0.028,0.042)	0.699
GP22	Frailty index	Inverse variance weighted	3	0.004(-0.034,0.042)	0.831
		Q-statistic			0.238
		Simple mode	3	-0.019(-0.076,0.037)	0.573
		Weighted mode	3	-0.019(-0.075,0.038)	0.589
		Weighted median	3	-0.004(-0.045,0.036)	0.829
		MR Egger	3	0.202(-0.040,0.444)	0.349
		egger_intercept			0.353
		MR-PRESSO	3	-	-
		global test			-
	Telomere length	Inverse variance weighted	3	-0.020(-0.058,0.018)	0.298
		Q-statistic			0.020
		Simple mode	3	-0.004(-0.046,0.039)	0.876
		Weighted mode	3	-0.010(-0.048,0.028)	0.671
		Weighted median	3	-0.015(-0.043,0.013)	0.292

		MR Egger	3	-0.049(-0.453,0.355)	0.853
		egger_intercept			0.912
		MR-PRESSO(Outlier-corrected)	3	-	-
		global test			-
GP23	Frailty index	Inverse variance weighted	4	0.011(-0.031,0.053)	0.600
		Q-statistic			0.015
		Simple mode	4	-0.009(-0.052,0.033)	0.701
		Weighted mode	4	-0.011(-0.043,0.022)	0.557
		Weighted median	4	-0.008(-0.037,0.022)	0.609
		MR Egger	4	-0.114(-0.263,0.034)	0.271
		egger_intercept			0.231
		MR-PRESSO	4	-0.005(-0.048,0.037)	0.820
		global test			0.065
	Telomere length	Inverse variance weighted	4	-0.002(-0.018,0.013)	0.761
		Q-statistic			0.270
		Simple mode	4	0.007(-0.021,0.035)	0.667
		Weighted mode	4	0.008(-0.014,0.029)	0.542
		Weighted median	4	0.004(-0.013,0.021)	0.634
		MR Egger	4	0.029(-0.042,0.101)	0.508
		egger_intercept			0.468
		MR-PRESSO(Outlier-corrected)	4	0.005(-0.009,0.020)	0.509
		global test			0.406
GP24	Frailty index	Wald ratio	1	-0.01(-0.039,0.019)	0.499
	Telomere length	Wald ratio	1	0.007(-0.010,0.024)	0.434

IgG, immunoglobulin G; GP, glycan peak; SNP, single-nucleotide polymorphism; CI, confidence intervals; IVs, instrumental variables; MR, Mendelian randomization; MR-PRESSO, Pleiotropy Residual Sum and Outlier; SNP, single nucleotide polymorphism.

Supplementary Table S3. Estimates of the causal effect of IgG N-glycosylation on the frailty index via multivariable MR

Exposure	Outcome	Method	Ivs	β(95%CI)	*p*
GP1				0.021(-0.071,0.113)	0.655
GP2				0.027(-0.106,0.160)	0.690
GP4				0.016(-0.264,0.296)	0.910
GP5				0.017(-0.112,0.146)	0.803
GP6				-0.036(-0.222,0.150)	0.704
GP7				-0.021(-0.239,0.197)	0.851
GP8				-0.057(-0.188,0.074)	0.391
GP9				-0.078(-0.190,0.034)	0.169
GP10				-0.011(-0.280,0.258)	0.938
GP11				-0.046(-0.189,0.097)	0.528
GP12				0.017(-0.175,0.209)	0.862
GP13	Frailty index	MV-IVW	56	-0.023(-0.146,0.100)	0.716
GP14				0.109(-0.152,0.370)	0.413
GP15				0.003(-0.326,0.332)	0.984
GP16				0.058(-0.069,0.185)	0.370
GP17				-0.041(-0.262,0.180)	0.720
GP18				-0.101(-0.315,0.113)	0.351
GP19				-0.056(-0.227,0.115)	0.517
GP20				-0.030(-0.171,0.111)	0.674
GP21				0.090(-0.108,0.288)	0.373
GP22				-0.043(-0.174,0.088)	0.525
GP23				-0.119(-0.219,-0.019)	0.019
GP24				0.081(-0.105,0.267)	0.392

IgG, immunoglobulin G; GP, glycan peak; SNP, single-nucleotide polymorphism; CI, confidence intervals; IVs, instrumental variables; MR, Mendelian randomization; IVW, inverse variance weighted; SNP, single nucleotide polymorphism.

Supplementary Table S4. Estimates of the causal effect of IgG N-glycosylation on telomere length via multivariable MR

Exposure	Outcome	Method	Ivs	β(95%CI)	*p*
GP1				0.037(-0.055,0.129)	0.433
GP2				0.140(0.020,0.260)	0.023
GP4				-0.153(-0.431,0.125)	0.281
GP5				-0.016(-0.139,0.107)	0.802
GP6				-0.013(-0.185,0.159)	0.886
GP7				-0.083(-0.308,0.142)	0.473
GP8				0.008(-0.121,0.137)	0.899
GP9				0.002(-0.112,0.116)	0.972
GP10				-0.027(-0.264,0.210)	0.823
GP11				0.009(-0.118,0.136)	0.889
GP12				-0.003(-0.193,0.187)	0.975
GP13	Telomere length	MV-IVW	56	-0.094(-0.210,0.022)	0.109
GP14				-0.089(-0.338,0.160)	0.481
GP15				0.038(-0.266,0.342)	0.806
GP16				-0.036(-0.169,0.097)	0.597
GP17				-0.024(-0.234,0.186)	0.819
GP18				0.011(-0.191,0.213)	0.913
GP19				0.015(-0.144,0.174)	0.852
GP20				0.138(-0.001,0.277)	0.052
GP21				-0.043(-0.261,0.175)	0.701
GP22				-0.027(-0.149,0.095)	0.661
GP23				-0.011(-0.111,0.089)	0.824
GP24				-0.013(-0.178,0.152)	0.878

IgG, immunoglobulin G; GP, glycan peak; SNP, single-nucleotide polymorphism; CI, confidence intervals; IVs, instrumental variables; MR, Mendelian randomization; IVW, inverse variance weighted; SNP, single nucleotide polymorphism.
